# Ionic Medication

**Published:** 1910-10-22

**Authors:** 


### Medicine.

### IONIC MEDICATION.

Ionic medication may be defined as the electrolytic dissociation of drugs by the galvanic current, the dissociated ions having the power to penetrate the tissues to a depth varying with the strength of current, time, and application, resistance of tissues, and the substance introduced. The field of its application is continually widening, and it seems likely to become a very useful branch of therapeutics. It should be one of the aims of medicine to replace general treatment as often as possible by local treatment, and to attain this ideal the electro-ionic method offers resources which no other method affords. It allows one to introduce medicaments into the cells which are impermeable to most remedies, and to obtain as many different actions as there are ions. It is now known that with prolonged application and with strong currents the ions can be made to penetrate to the deeper parts so as to produce definite and practical results.

Ionic medication has obvious advantages over the system of giving large doses by the mouth in that the effects of the drugs are to a certain extent limited to the parts that require treatment instead of the whole, as well as the diseased tissues, being subjected to their action. It has been found that in the introduction by electrolysis of cocaine for local anæsthesia, the latter is more durable than when cocaine is hypodermically injected, though the amount of the drug used is less, and there is, moreover, no absorption into the general circulation. It has also been found that when lithium is introduced by electrolysis its elimination by the urine continues for a longer period than when taken by the mouth. Moreover, the action of the ions is not always the same as that of the elements in their molecular conditions, for free chlorine is a strong oxydising body, whilst chlorine ions have no such protoplasm-destroying properties; and the same may be said for iodine.

### Methods of Ionic Medication.

Ionic medication for ordinary purposes can be carried out effectively by Leclanché battery cells capable of driving 30 to 40 milliamperes through the human body, a milliamperemeter to read the current, and suitable electrodes and connecting wires. The treatment should be almost painless, provided the current is turned off gradually. There should be no pain at all during the passage of the continuous current. It is, of course, of the very greatest importance to ascertain which is the positive and which is the negative pole, for some ions are given off by the positive and some by the negative. One way is to have some pole-finding paper, which, when moistened, turns scarlet where acid is formed around the negative pole. Another simple way is to put both terminals into a vessel of water; bubbles of gas will be given off by the negative pole when the current is turned on. The anode is the pole at which acids are liberated, particularly oxygen and hydrochloric acid from the tissues, and it is, relatively speaking, the sedative pole. At the kathode alkalies, chiefly caustic soda and hydrogen, are liberated, and it is a stimulant pole. Kations are ions which are introduced under the anode, being substances which are transmitted by the electrical current in the direction of the kathode, and they include the hydrogen ions of acid substances, the metallic ions of salts of copper, and so forth, the ions of basic radicles such as ammonia and alkaloids in the case of cocaine, morphia, etc. Anions are substances which are introduced beneath the kathode, and they include the acid radical ions such as $SO_4$, bromides, iodides, and the hydroxyl ions of alkalies. For instance, in epilation of superfluous hairs by means of an electric current and a steel needle, it is most important that the latter should be attached to the negative pole, for if it were attached to the positive pole the iron would be carried into the hair follicles, leaving a mark. As a matter of fact, it is unusual to use iron electrodes, but rather to employ insensitive electrodes or sensitives, such as copper and zinc, or electrolytic solutions such as salts, acids, or bases.

The diffusion of the different ions in the tissues varies very much owing to reasons which vary in different cases. Thus zinc is not absorbed from the site of local application owing to the coagulation of albumin around it. Cocaine becomes introduced into the plasma of the cells, and stays there so as not to become further absorbed. Strychnine and prussic acid rapidly diffuse, and cause death in a few minutes. The dosage varies principally with the time of application and the strength of current, but the resistance of the tissues and the substance introduced are also important factors to be considered. The smaller the atomic weight the quicker the ion travels. At present the dosage is not definitely fixed, though, as in the case of the $x$-rays, this initial difficulty will presently be overcome when further experience has been gained.

In ionic medication different effects may be produced according as a large or small dose is employed. Thus zinc in small doses causes irritation of the skin and promotes the growth of hair, but in large doses it destroys the tissues. As a general rule it may be stated that a 2-per-cent. solution of copper sulphate with a current of three milliamperes per square centimetre will allow of its being introduced for the desired depth in a case of lupus erythematosus, for instance, in 15 minutes. A 10-per-cent. solution of cocaine with a current of 10 milliamperes per square centimetre will cause complete local anæsthesia of the skin in ten minutes.

Two other conditions in which this form of treatment has proved eminently successful are, first, neuralgia and pain in the nerves, of the nature, for instance, of tic douloureux; and, secondly, stiffness of joints. In the treatment of severe neuralgia several layers of lint soaked in a 2-per-cent. solution of sodium salicylate should be placed over the seat of the pain and connected with the negative pole of the battery for about 20 minutes; when neuralgia is in the region of the head it is particularly important that the current should be altered very gradually, as severe vertigo may temporarily ensue. Perhaps the most brilliant results yet recorded have been in connection with stiff joints. The treatment can be employed with a 1 or 2 per cent. solution of sodium chloride either in a bath or with several layers of lint saturated with the solution, according to the situation of the joint affected. A current of the strength of 10 milliamperes may be more than can be borne at first, but after about a quarter of an hour a much greater current will be tolerated. One precaution that is particularly needed is that there should be no excoriation of the skin, or troublesome and painful burns are apt to result. If during the treatment the patient complains of a burning pain the current should be stopped, and the places in which the pain has been complained of wiped with pure alcohol and covered with flexile collodion. For stiffened joints a 2-per-cent solution of sodium chloride attached to the negative pole with a current of 30 milliamperes for 30 minutes thrice weekly is the treatment adopted. Iodine is also employed in the same way, especially after septic affections of joints. It can be used as an aqueous solution in a bath or on lint, and if need be the colourless variety can be employed. Iodine is more caustic than sodium chloride, and so the séance should be shorter with the latter, and the current should be less.

